# Transcriptomic analysis identifies diagnostic genes in polycystic ovary syndrome and periodontitis

**DOI:** 10.1186/s40001-023-01499-4

**Published:** 2024-01-02

**Authors:** Xiaodan Liu, Jingran Zhang, Xiao Wang, Zhihui Zhang

**Affiliations:** 1 Department of Stomatology, Peking University Third Hospital, Beijing, China; 2 Department of Periodontology, Peking University School and Hospital of Stomatology & National Center of Stomatology & National Clinical Research Center for Oral Diseases & National Engineering Laboratory for Digital and Material Technology of Stomatology & Beijing Key Laboratory of Digital Stomatology & Research Center of Engineering and Technology for Computerized Dentistry Ministry of Health & NMPA Key Laboratory for Dental Materials, Beijing, China

**Keywords:** Polycystic ovary syndrome, Periodontitis, GSEA, Diagnosis, Transcriptomic analysis

## Abstract

**Purpose:**

To investigate underlying co-mechanisms of PCOS and periodontitis through transcriptomic approach.

**Methods:**

PCOS and periodontitis gene expression data were downloaded from the GEO database to identify differentially expressed genes. GO and KEGG pathway enrichment analysis and random forest algorithm were used to screen hub genes. GSEA analyzed the functions of hub genes. Correlations between hub genes and immune infiltration in two diseases were examined, constructing a TF-ceRNA regulatory network. Clinical samples were gathered from PCOS and periodontitis patients and RT-qPCR was performed to verify the connection.

**Results:**

There were 1661 DEGs in PCOS and 701 DEGs in periodontitis. 66 intersected genes were involved and were enriched in immune and inflammation-related biological pathways. 40 common genes were selected from the PPI network. RF algorithm demonstrated that ACSL5, NLRP12, CCRL2, and CEACAM3 were hub genes, and GSEA results revealed their close relationship with antigen processing and presentation, and chemokine signaling pathway. RT-qPCR results confirmed the upregulated gene expression in both PCOS and periodontitis.

**Conclusion:**

The 4 hub genes ACSL5, NLRP12, CCRL2, and CEACAM3 may be diagnostic genes for PCOS and periodontitis. The created ceRNA network could provide a molecular basis for future studies on the association between PCOS and periodontitis.

**Supplementary Information:**

The online version contains supplementary material available at 10.1186/s40001-023-01499-4.

## Introduction

Polycystic ovary syndrome (PCOS) is a complex genetic condition with 5% to 15% prevalence globally. PCOS is a highly prevalent heterogeneous syndrome of clinical and/or biochemical androgen excess, ovulatory dysfunction, and polycystic ovaries (PCO) [1]. In addition, PCOS is associated with psychological effects such as depression, anxiety, and reduced quality of life. Women with PCOS have an increased risk of developing hormonal problems, and two-thirds of them have metabolic dysfunction, which increases the risk of developing type 2 diabetes mellitus (T2DM) and cardiovascular diseases, and appears to be a complex trait which results from genetic and environmental influences [1, 2].

Periodontitis is characterized by pathological loss of periodontal ligament and alveolar bone, which involves dynamic interactions among bacteria and destructive immune responses [3]. Besides, it is a non-communicable disease characterized by gingival inflammation as well as exacerbated and uncontrolled inflammatory response. When destruction of the periodontium is chronically exposed to microbial dysbiosis, periodontal bacteria may evolve to evade host immune responses through ulcerated epithelium and destruction of the periodontium. This process enhances inflammatory aspects, which probably leads to an adverse effect on systemic health [4].

Some published literatures have mentioned there is an association between PCOS and periodontitis [5, 6]. For instance, previous studies demonstrated that PCOS patients may be more inclined to develop periodontitis [7–9]. In addition, a recent meta-analysis showed that females with PCOS have a higher risk of periodontitis, and vice versa [10]. Besides, periodontitis patients with PCOS were more prone to severe clinical symptoms such as deeper periodontal pocket, gingival bleeding, and clinical attachment loss compared to those without PCOS [10]. Although the previous findings have demonstrated the clinical feature and laboratory indicators relationship between PCOS and periodontitis, and early diagnostic genes in PCOS or periodontitis alone have been well characterized [11–14], data on genetic-level association between the two diseases remain scant.

In this study, we hypothesized that there could be similar transcriptomic alterations in periodontitis and PCOS. To verify this hypothesis, we profiled the gene expression from the relevant datasets and identified known genes from the database. We then carried out a detailed bioinformatics analysis to define key genes, biological processes and signaling pathways that may serve as molecular links between periodontitis and PCOS. These data yielded a comprehensive understanding of pathophysiological relationships between the two diseases, and laid the basis for discovery of targets for risk assessment.

## Materials and methods

### Data sources and batch effect correction

GSE106724 and GSE137684, which contained mRNA sequencing data of ovarian granulosa cells, and GSE173078 and GSE79705, including female gingival tissues mRNA sequencing data, were downloaded from the GEO database (http://www.ncbi.nlm.nih.gov/geo). To be more specific, there were 8 PCOS and 4 controls in GSE106724, 8 PCOS and 4 controls in GSE137684. Additionally, 6 periodontitis and 2 controls, and 6 periodontitis and 10 controls were in GSE70795 and GSE173078, respectively. Since the number of PCOS or periodontitis data in each data set was small, the data of GSE106724 and GSE137684 were combined and regarded as training set 1 for PCOS correlation analysis. In addition, the data of GSE173078 and GSE79705 (only female samples were selected) were combined and considered as training set 2 for the correlation analysis of periodontitis. Then, the batch effect among the data sets was presented into a principal component analysis (PCA) diagram and, and the batch effect was corrected by ComBat in sva package (version 3.38.0).

### Identification and functional analysis of common genes in PCOS and periodontitis

Differentially expressed genes (DEGs 1) between 16 PCOS and 8 control samples and the DEGs 2 between 12 periodontitis and 12 control samples were identified from training set 1 and training set 2 individually by Limma R package (version 3.46.0) with the thresholds of |log_2_FC|> 0.5 and *p*-value < 0.05, and the results were visualized into heatmaps by ggplot2 (version 3.3.3). In order to ensure the consistent expression trend of genes in the two diseases, genes with the same expression trend were overlapped between DEGs 1 and DEGs 2 to obtain intersected genes by VennDiagram (version 1.6.20) in R package.

In order to investigate whether there was an interaction between the intersected genes, STRING (https://string-db.org) was used to construct a protein–protein interaction (PPI) network for these genes with confidence = 0.4 as the standard, and the intersected genes in the network were considered as common genes. The topology characteristics of PPI network were analyzed by CytoHubba tool in Cytoscape.

In addition, Gene Ontology (GO) and Kyoto Encyclopedia of Genes and Genomes (KEGG) enrichment analyses were employed on the common genes using ClusterProfiler R package (version 1.10.2). The enriched items could meet count ≥ 1 and *p*-value < 0.05 were considered as significantly enriched. Then, the significantly enriched KEGG pathways and the corresponding genes were extracted to build a common gene-pathway network which was visualized by Cytoscape (version 3.8.2).

### Identification of potential diagnostic genes for PCOS and periodontitis

We employed the caret R package to perform random forest (RF) algorithm on the common genes in the training set 1 and training set 2, respectively. The plot function in DALEX R package was used to visualize the distribution of residuals for the random forest model. The variable important plot was used to show the relative importance of each common gene in predicting the response. Candidate hub genes 1 and candidate hub genes 2 were selected by whether the relative importance was greater than the group score in each training set. The hub genes were then obtained by overlapping candidate hub genes 1 and 2.

Receiver operating characteristic (ROC) curves of each hub gene were generated to assess their diagnostic value. Hub genes with an area under the curve (AUC) > 0.7 were considered to have high accuracy in diagnosis and were identified as diagnostic genes for PCOS and periodontitis. Simultaneously, Pearson correlation analysis was employed to the diagnostic genes and boxplots of the genes were also plotted to visualize their expressions in normal PCOS, and periodontitis samples. Then, the co-expression network of the diagnostic genes was constructed through an online database, GeneMANIA (http://genemania.org/). In addition, Gene Set Enrichment Analysis (GSEA) of each diagnostic genes were performed using KEGG gene sets downloaded from MsigDB as a reference to investigate the possible mechanisms of diagnostic genes involved in PCOS and periodontitis.

### Explore the correlation between diagnostic genes and immune infiltration

Mcp_counter algorithm in immunedeconv package (version 1.0.4) was used to calculate the proportion of 10 immune cells (T cell, T cell CD8+, cytotoxicity score, NK cell, B cell, monocyte, macrophage/monocyte, myeloid dendritic cell, neutrophil and endothelial cell) in PCOS and periodontitis using the Immunedeconv package (Version 2.0.4), and the results were output as heatmaps by pheatmap (version 1.0.12).

Then, corrplot (version 0.91) was applied to plot the correlations between the 10 immune cells obtained through Pearson correlation analysis. Furthermore, rank-sum test was utilized to compare the differences in immune cell between disease samples and normal samples. Finally, the Pearson analysis was further performed to analyze the correlations between diagnostic genes and the 10 immune cells.

### Prediction of transcription factors, miRNAs, lncRNAs, and drugs targeting the diagnostic genes

NetworkAnalyst (https://www.networkanalyst.ca/) was employed to predict the transcription factors (TFs) binding and regulating the expression of diagnostic biomarkers. The prediction was performed under the following settings: peak intensity signal < 500 and the predicted regulatory potential score < 1. Moreover, the miRNAs targeting diagnostic biomarkers were predicted using the miRWalk with bindingp = 1 and energy < − 25. Next, lncRNAs interacting with the predicted miRNAs with low stringency ≥ 1 were identified using the Starbase. The interactions were integrated and visualized into a TF-ceRNA network by the Cytoscape software. In addition, the Drug Bank (https://go.drugbank.com/) was used to screen candidate drugs targeting the diagnostic genes for potential treatment of PCOS and periodontitis patients.

### Clinical analysis: real-time quantitative PCR

Abandoned oocytes samples were gathered from six female patients who received infertility treatments in Peking University Third Hospital Reproductive Medicine Center.

Three of the patients suffered from PCOS (according to 2003 ESHRE/ASRM diagnostic criteria), and the others were fallopian tube infertility, and they were all periodontal healthy. Six systemic healthy female patients were recruited from Peking University Third Hospital Stomatology Department with abandoned gingiva samples. Three of them were diagnosed of periodontitis (according to 2019 diagnostic criteria) and received periodontal flap surgery, and the other three were periodontal healthy but an impacted 3rd molar needed extraction. This study was approved by Peking University Third Hospital Medical Science Research Ethics Committee (M2022106), and written informed consents were received from all participants for their enrollment. Quantitative reverse transcription polymerase chain reaction (RT-qPCR) experiments were performed using a total of six oocytes samples and six gingiva samples. The total RNA of 12 samples were isolated by the TRIzol Reagent following the manufacturer's guidance (Ambion, USA). Next, total RNA were inversely transcribed into cDNA utilizing the SweScript-First-strand-cDNA-synthesis-kit (Servicebio, China), according to the manufacturer's protocol. QPCR was subsequently performed using the 2xUniversal Blue SYBR Green qPCR Master Mix (Servicebio, China). The primer sequences for PCR are displayed in Additional file 6: Table S1. The relative gene expression level was uniformized to the internal reference ACTIN and calculated using the 2^−ΔΔCq^ method.

## Results

### Identification and functional analysis of common genes involved in PCOS and periodontitis

**Fig. 1:**
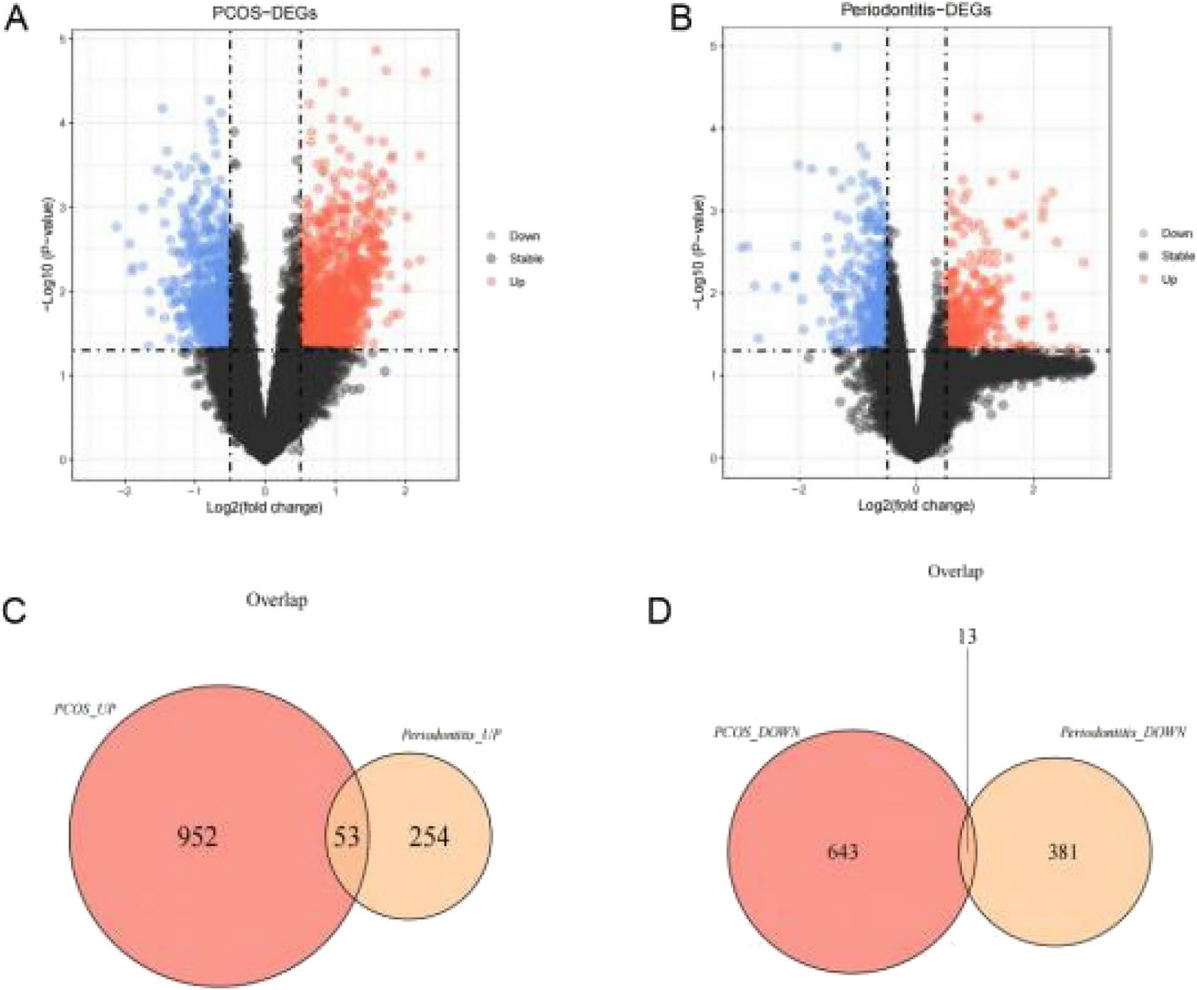
Identification of common genes involved in PCOS and periodontitis. **A** Volcano plot of differentially expressed genes in training set 1; **B** volcano plot of differentially expressed genes in training set 2; **C** Venn diagram of co-unregulated genes of training set 1 and training set 2; **D** Venn diagram of co-downregulated genes of training set 1 and training set 2

It was obvious that there were batch effects existing between the data from different sequencing platforms, and the data tended to cluster after batch effect correction by ComBat (Additional file 1: Fig. S1). To identify common genes involved in PCOS and periodontitis, we compared the gene expression profile between disease and control samples in the two training datasets. A total of 1,661 DEGs 1(Fig. 1A) and 701 DEGs 2 (Fig. 1B) were identified from each training dataset. Next, a total of 66 intersected genes (Additional file 7: Table S2), which included 53 upregulated (Fig. 1C) and 13 downregulated genes (Fig. 1D), were obtained after the overlap analysis.

After the interaction analysis of the 66 intersected genes by STRING, a PPI network of 40 interaction pairs was established, which included 126 nodes and 126 edges (Additional file 2: Fig. 2A, Additional file 8: Table S3). Therefore, the 40 intersected genes in the PPI network were regarded as common genes for the subsequent analyses. Through GO and KEGG enrichment analyses, the 40 common genes were significantly enriched in 93 BPs, 2 CCs, 5 MFs, and 19 KEGG pathways (Additional file 9: Table S4 and Additional file 10: Table S5). The genes were mainly involved in immune and inflammation-related biological functions, such as neutrophil activation involved in immune response, neutrophil degranulation (Additional file 2: Fig. S2B) and pathways such as neutrophil extracellular trap (NET) formation, Fc epsilon RI signaling pathway and natural killer cell-mediated cytotoxicity (Additional file 2: Fig. S2C). The gene regulation network was constructed including 24 common genes, 63 interaction pairs, and 10 KEGG pathways (Additional file 2: Fig. S2D).

**Fig. 2:**
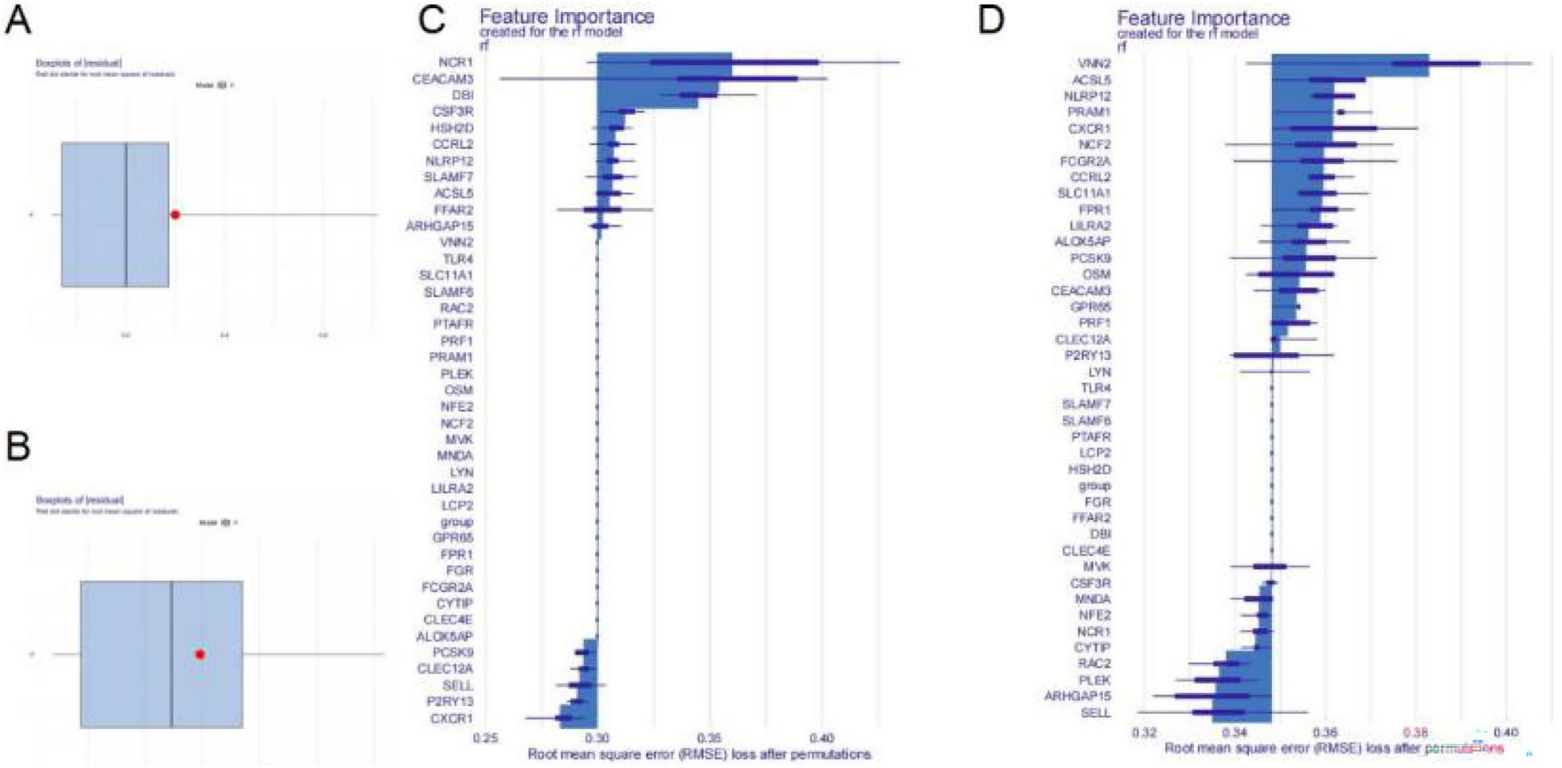
Genes had significant contributions to the RF models. **A** Boxplot describes the residuals within a cluster in PCOS; **B** boxplot describes the residuals within a cluster in periodontitis; **C** feature importance of genes to the RF models in PCOS; **D** feature importance of genes to the RF models in periodontitis; the horizontal axis of both graphs represents RMSE loss after permutations. The vertical axis is gene names

### Identification of hub genes in PCOS and periodontitis

Thereafter, RF algorithm was performed in the training set 1 (Fig. 2A) and training set 2 (Fig. 2B). Analysis of variables showed that 11 genes (ARHGAP15, FFAR2, ACSL5, SLAMF7, NLRP12, CCRL2, HSH2D, CSF3R, DBI, CEACAM3 and NCR1) had significant contribution to the RF model in PCOS (Fig. 2C) which were candidate hub genes 1, and 20 genes (LYN, P2RY13, CLEC12A, PRF1, GPR65, CEACAM3, OSM, PCSK9, ALOX5AP, LILRA2, FPR1, SLC11A1, CCRL2, FCGR2A, NCF2, CXCR1, PRAM1, NLRP12, ACSL5, and VNN2) had the highest effect in the RF model in periodontitis (Fig. 2D) which candidate hub genes 2. Intersection of the candidate genes 1 and 2 yielded 4 hub genes (ACSL5, NLRP12, CCRL2, and CEACAM3) involved in both PCOS and periodontitis.

### Evaluation of the diagnostic value of the hub genes in PCOS and periodontitis

Next, the diagnostic values of 4 hub genes in PCOS and periodontitis were explored. In the PCOS training dataset, the AUCs of ACSL5, NLRP12, CCRL2, and CEACAM3 were 0.828, 0.820, 0.719 and 0.867, respectively (Additional file 3: Fig. S3), indicating their performances in distinguishing PCOS from control samples were great. Additionally, the AUCs of ACSL5, NLRP12, CCRL2, and CEACAM3 in the periodontitis training dataset were 0.771, 0.819, 0.708 and 0.785, respectively, which also suggested the diagnostic values of 4 hub genes on periodontitis were strong (Additional file 4: Fig. S4). As a result, the 4 hub genes could be considered as diagnostic genes for both PCOS and periodontitis.

### Functional analysis of the diagnostic genes

**Fig. 3:**
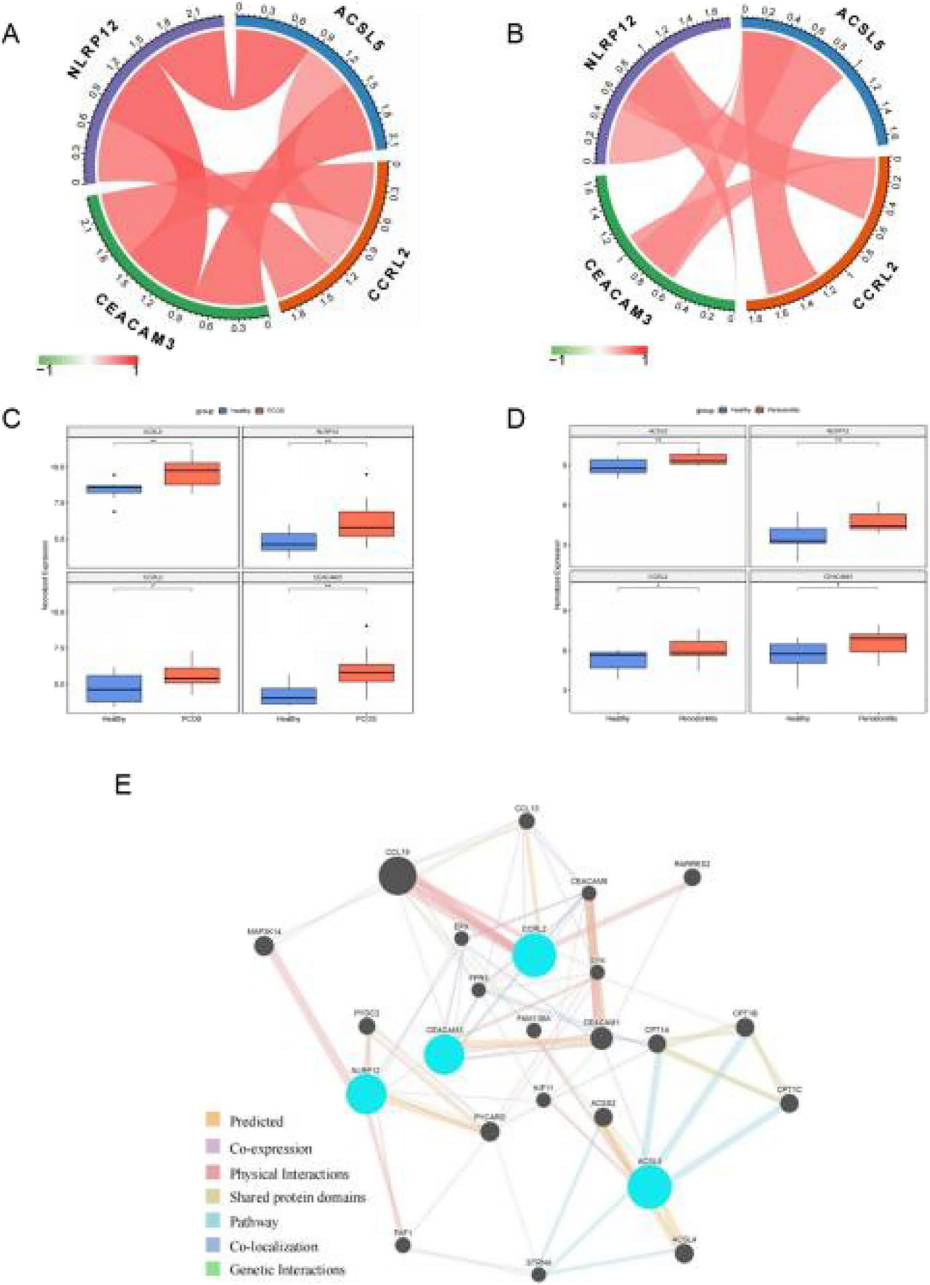
Correlation networks and expressions of hub genes in PCOS and periodontitis, and co-expression network of hub genes. **A** Interactions between hub genes in PCOS training set; **B** interactions between hub genes in periodontitis training dataset; **C** expression of hub genes between healthy and PCOS samples. Red and blue represent PCOS and healthy samples, respectively; **D** expression of hub genes between healthy and periodontitis samples. Red and blue represent periodontitis and healthy samples, respectively; **E** co-expression network of hub genes

**Fig. 4:**
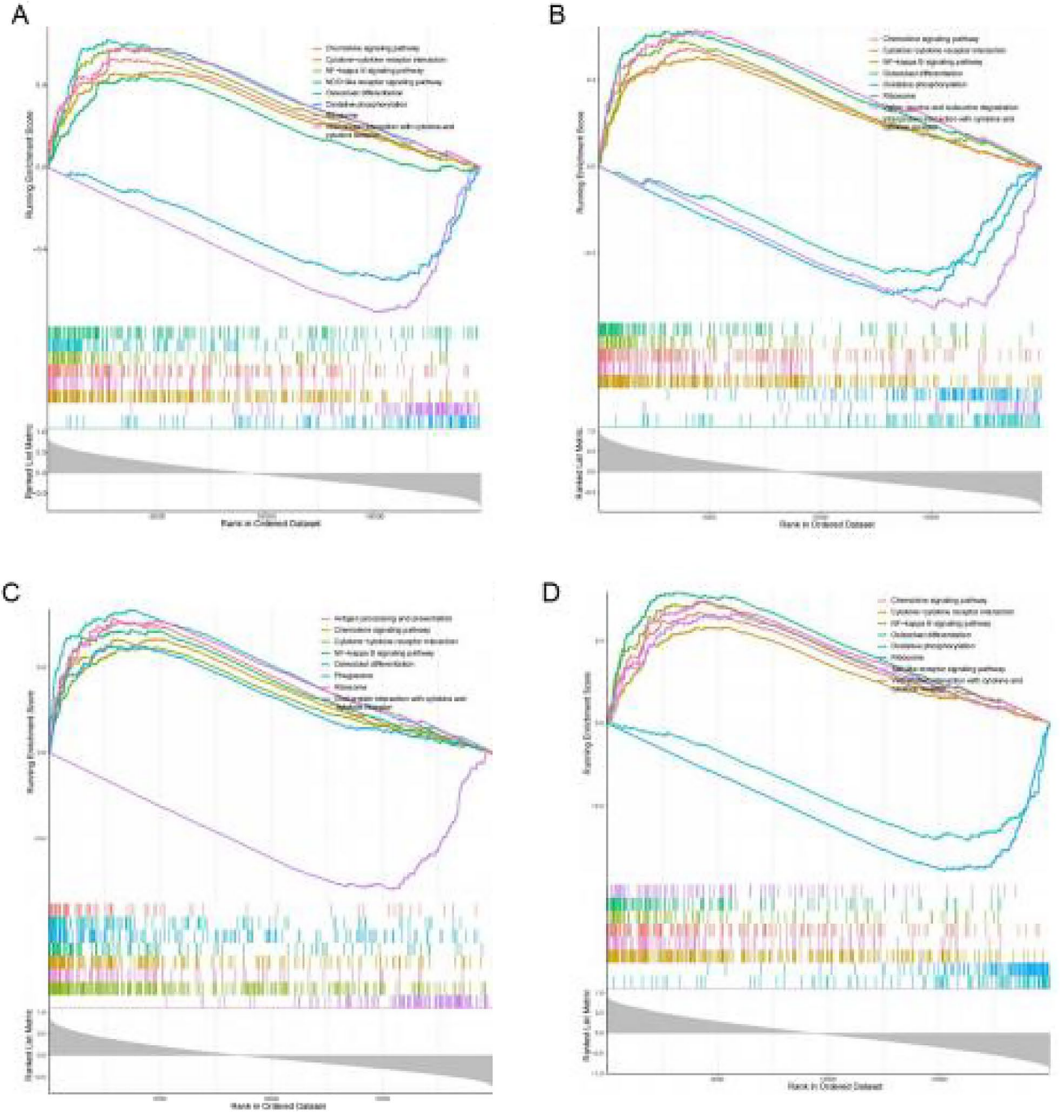
GSEA enrichment analysis of hub genes. **A** KEGG pathway analysis of hub gene ACSL5; **B** KEGG pathway analysis of hub gene NLRP12; **C** KEGG pathway analysis of hub gene CCRL2; **D** KEGG pathway analysis of hub gene CEACAM3

The Pearson correlation analysis results illustrated the 4 diagnostic genes had strong positive correlations with each other in both PCOS and periodontitis (Fig. 3A, B). Moreover, the expressions of these 4 genes were all upregulated in PCOS and periodontitis (Fig. 3C, D). It also can be observed that the 4 diagnostic genes may share protein domains, have physical interactions, and participate in the same pathway with other genes (Fig. 3E). The GSEA results demonstrated that the diagnostic genes were related to many KEGG pathways associated with chemokine signaling pathway, cytokine–cytokine receptor interaction, NF-kappa B signaling pathway, NOD-like receptor signaling pathway, osteoclast differentiation, viral protein interaction with cytokine and cytokine receptor (Fig. 4A–D).

### Association between diagnostic genes and immune

**Fig. 5:**
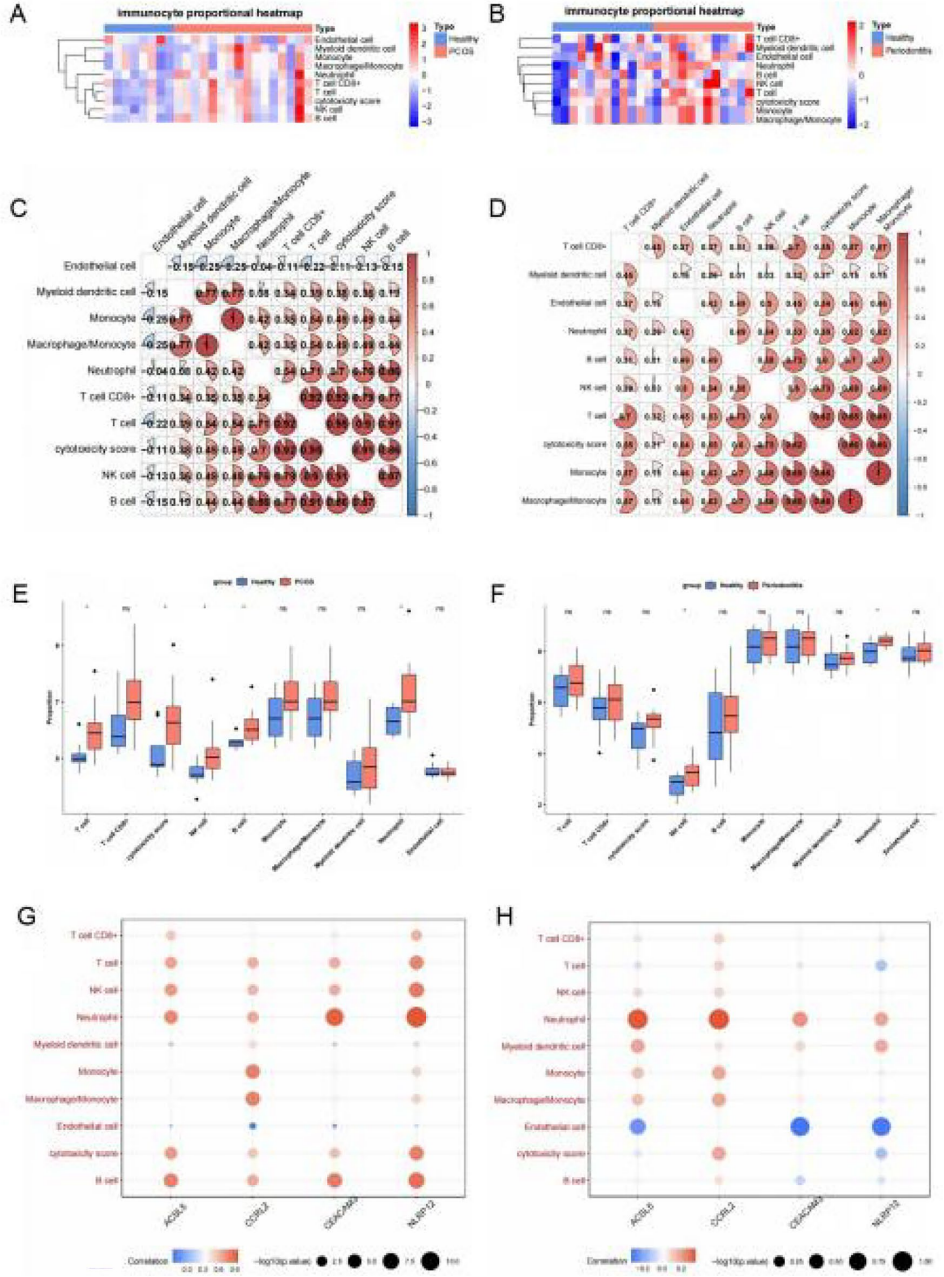
The correlation between the hub gene and immune infiltration. **A** Immunocyte proportional heatmap of 10 immune cells between healthy and PCOS samples; **B** immunocyte proportional heatmap of 10 immune cells between healthy and periodontitis samples; **C** correlations between immune cells in PCOS; **D** correlations between immune cells in periodontitis; **E** proportion differences of immune cells between healthy and PCOS samples; **F** proportion differences of immune cells between healthy and periodontitis samples; **G** correlations between immune cells and diagnostic genes in PCOS; **H** correlations between immune cells and diagnostic genes in periodontitis

In order to investigate the association between immune cells and the 4 diagnostic genes, the proportions of the 10 immune cells were computed in diseases samples and normal samples. It can be seen that, overall, the proportions of 10 immune cells were significantly higher in both PCOS and periodontitis samples compared with normal samples (Fig. 5A, B). In addition, there was a positive correlation between each of the 10 immune cells in both diseases (Fig. 5C, D). The boxplots revealed that the proportions of T cell, cytotoxicity score, NK cell, B cell, and neutrophil were higher in PCOS, and the proportions of NK cell and neutrophil were higher in periodontitis, compared to normal samples (Fig. 5E, F). Finally, there was a strong positive correlation between diagnostic genes and immune cells in PCOS. Nevertheless, in periodontitis, the correlation between diagnostic genes and immune cells was not obvious (Fig. 5G, H).

### TF-ceRNA network prediction

In addition, the TFs prediction results showed that several target TFs could be predicted only by ACSL5 and CCRL2 (Additional file 5: Fig. S5A). Besides, 21 miRNAs and 178 lncRNAs were shown to potentially affect the expression of ACSL5, NLRP12, CCRL2, and CEACAM3, and their ceRNA network was built (Additional file 11: Table S6 and Additional file 5: Fig. S5B). After integrating the predicted TFs with the ceRNA, a TF-ceRNA network was constructed (Additional file 5: Fig. S5C).

### Verification of the expression of inflammation-associated biomarkers in clinical samples

**Fig. 6:**
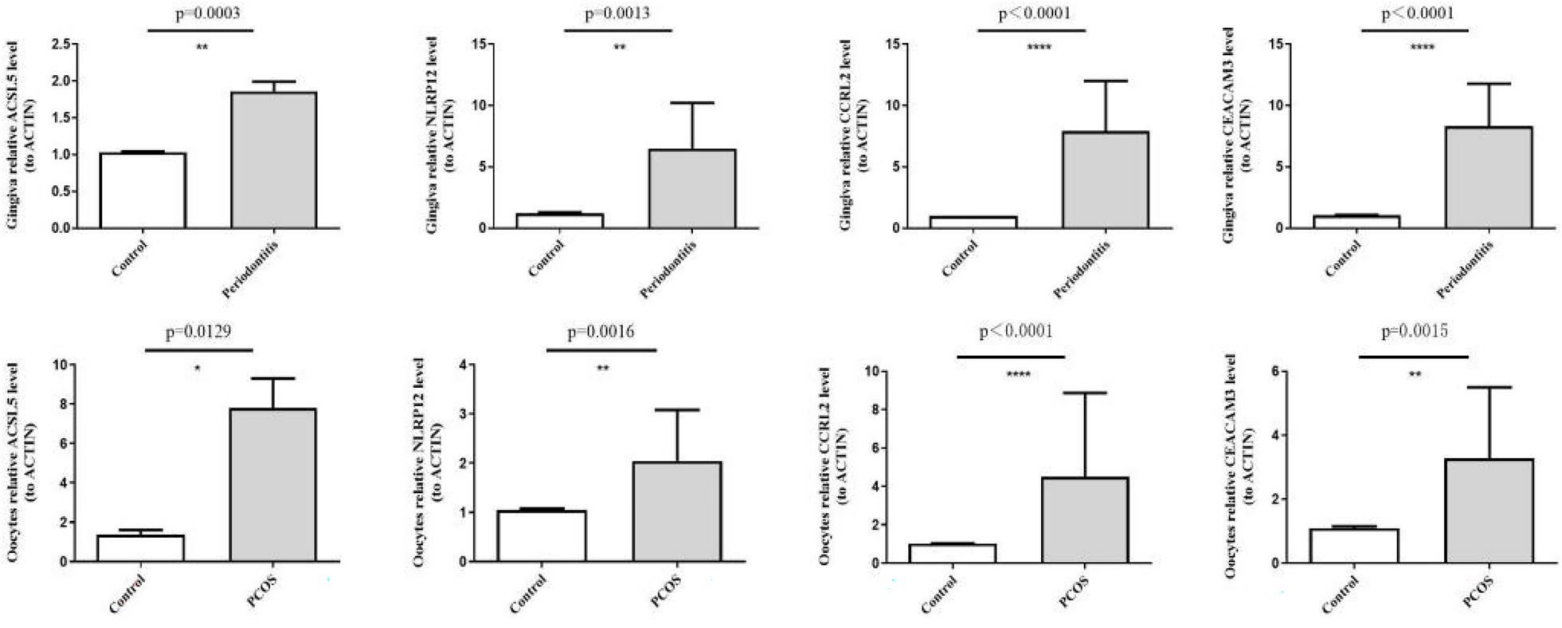
The expression levels of four hub genes in PCOS oocytes and periodontitis gingiva samples were detected by RT-qPCR compared to controls. *p < 0.05, **p < 0.01, ***p < 0.001, ****p < 0.0001

All four inflammation-associated biomarkers were upregulated in PCOS oocytes samples compared to controls, and they were the same in gingiva samples. In agreement with the results of the public database data analysis, four genes were significantly more highly expressed in both PCOS oocytes and periodontitis gingiva compared to healthy controls (*p* < 0.05) (Fig. 6).

## Discussion

Our study demonstrated that there may be common patterns in the expression of genes involved in the immune processes of PCOS and periodontitis. We identified a total of 40 common genes in PCOS and periodontitis, which were enriched in immunological and inflammation-related biological pathways. ACSL5, NLRP12, CCRL2, and CEACAM3 were identified as hub genes and diagnostic genes in both PCOS and periodontitis.

GO and KEGG analysis revealed that the common genes were significantly enriched in 93 BPs, 2 CCs, 5 MFs and 19 KEGG pathways. Besides, the genes were mainly involved in immune and inflammation-related biological functions, such as neutrophil activation involved in immune response, neutrophil degranulation and pathways such as neutrophil extracellular trap (NET) formation, Fc epsilon RI signaling pathway and natural killer cell-mediated cytotoxicity.

Neutrophils, as the most abundant leukocytes in blood, are regarded as the first line of defense during infection and inflammation [15]. When microorganisms infest the body, they can trigger an inflammatory response, which recruits neutrophils from the circulation into the tissues. Neutrophils can destroy microorganisms by phagocytosis and the formation of neutrophil extracellular traps (NETs) [16]. Additionally, the activated neutrophils release proteinases into surrounding tissues, causing damage to them. Leukocytes, along with granulosa cells, may contribute to PCOS by secreting cytokines implicated in follicle growth. A previous study showed that women with PCOS had significantly higher inflammatory-immune markers in their peripheral blood, and the number of neutrophils was higher in PCOS women than in controls, the presence of CD4+ T cells and NK cells was an independent risk factor for PCOS and contributed to its pathogenesis. It is also found that lower neutrophil gelatinase-associated lipocalin (NGAL) concentrations in PCOS women, may activate atherotic plaque erosion [17].

The initial cause of periodontitis is thought to be dysbiosis, an imbalance in bacteria in the oral microbiota [18]. Bacterial biofilm accumulation causes an increase in the inflammatory infiltrates, which are primarily composed of neutrophils, within oral tissues. The neutrophils form a barrier in the deeper periodontal tissues to prevent bacteria from invading. Hence, a lack of neutrophils results in severe periodontitis. People with defects in the production and distribution of neutrophils are at risk of severe periodontitis. Some of these defects are congenital and rare such as Chediak-Higashi syndrome, Papillon–Lefèvre syndrome, neutropenias, and leukocyte adhesion deficiency [19, 20]. It is also possible to develop severe periodontitis despite neutrophilia, as a result of a lack of neutrophil control of bacterial infection [21]. More neutrophils with a prosurvival phenotype are present in patients with severe forms of periodontitis. Periodontal pathogens such as *Porphyromonas gingivalis* possess a survival mechanism that prevents microbial killing and promotes inflammation. On the other side, inflamed periodontal tissues contain a large number of neutrophils, which correlates with the severity of the lesions, and the tissue destruction may be a collateral effect of hyperactive neutrophils [22, 23]. In addition, a previous study demonstrated that salivary neutrophil elastase (NE) level was higher in the systemically healthy women with gingivitis compared with PCOS women with periodontally healthy, which revealed that NE level may pertain to both PCOS and periodontitis.

Through RF of the identified hub genes (ACSL5, NLRP12, CCRL2, and CEACAM3), pathways that activate the immune system were implicated in PCOS and periodontitis. ACSL5, one of the members of Acyl-CoA synthetase long-chain (ACSL) proteins family, plays a crucial role in fatty acid metabolism and is widely studied for its presence in tissues such as brown adipose tissue, skeletal muscle, liver, and brain [24]. The metabolic consequences of obesity include insulin resistance and compensatory hyperinsulinemia, which decrease lipolysis and increase adipogenesis. It is well known that obesity is associated with either PCOS or periodontitis [25, 26]. In comparison to age-matched and BMI-matched controls, women with PCOS tend to have visceral adiposity. Excess weight in PCOS amplifies adverse metabolic outcomes, and increase the relative risk of T2DM [27], and even lead to more adverse metabolic and reproductive outcomes. Periodontitis has been suggested to be linked to obesity and metabolic syndrome, and the evidence from epidemiological studies supports the association between these conditions. A meta-analysis of 26 studies found an odds ratio of 1.38 (95% CI 1.26–1.51) between metabolic syndrome and periodontitis, the risk of periodontitis was 38% higher for individuals with metabolic syndrome [28]. The underlying biological mechanism for this association involves cytokines such as tumor necrosis factor-α and interleukin-6, produced by adipose tissue, which affect the whole body and contribute to development of a low-grade systemic inflammation [26]. NLRP12 is a member of the NOD-like receptors (NLRs) family, which inhibits inflammation and osteoclast genesis by inhibiting the NF-κB pathway, contributes to the prevention of periapical bone destruction in mice [29]. There is a strong expression of NLRP12 in gingival tissues with severe chronic periodontitis [30, 31]. Although the current study did not directly reveal the role of NLRP12 in PCOS, a study in PCOS mice model mentioned hyperandrogen stimulates chronic low-grade inflammation in the ovary to activate the NLRP3 (another member of the NLR family) inflammasome, further inducing a series of pathologies including ovarian GC pyroptotic death, follicular dysfunction and ovarian interstitial cell fibrosis. C–C chemokine receptor-like 2 (CCRL2) acts as a presenting molecule that mediates the formation of non-soluble chemotactic gradients for leukocytes expressing CMKLR1 (the functional chemerin receptor), or form heterocomplexes with other chemokine receptors, so that it can modulate leukocyte migration. Chemerin is a chemoattractant protein that directs inflammatory cells that express ChemR23 (its receptor) towards sites of inflammation. Ozcan et al. found compare with healthy controls, the significantly higher expression of chemerin and ChemR23, and slightly higher levels of CCRL2 expression in periodontitis tissues [32]. Recent animal studies also demonstrated the expression of CCRL2 was also drastically reduced granulosa cells of periovulatory follicles and corpora lutea of mice [33], and higher transcript content of CCRL2 was observed in large follicles of mature gilts, whereas the lower mRNA level was noted in medium follicles of mature gilts [34]. Carcinoembryonic antigen-related cell adhesion molecule 3 (CEACAM3) is mainly expressed in the lung, and also be reported to be localized in immune cells at the cervical stroma and inflammatory bowel diseases (IBD) [35–38]. Since periodontitis is an inflammatory disease, and the occurrence of PCOS will increase the risk of endometrial cancer, CEACAM3 may play important roles in the development of PCOS and periodontitis, but the specific role needs to be further studied. Of course, detailed mechanism needing to be further investigated.

According to our research, it was the first time to employ bioinformatics tools to analyze gene co-expression networks between PCOS and periodontitis. Although our study highlights important findings, there are still several limitations in this study. Firstly, the included data were generated from publicly available micro-array/RNA sequencing analyses of periodontitis and PCOS cases from different population sources. Secondly, the study lacks detailed data on patients with both periodontitis and PCOS, thus future studies should validate these findings in cohorts with both diseases. Overall, our study identified putative significant crosstalk genes involved in molecular mechanisms in PCOS and periodontitis. The purported linkage genes were validated experimentally, and also existing evidence generally supported our findings. These findings from experimental genomics may provide well-supported hypotheses, which could be used as a basis for future research on the pathophysiology of PCOS–periodontitis interactions.

## Conclusion

In our study, we analyzed transcriptome genes from PCOS and periodontitis and identified crosstalk genes, modules, and biochemical processes and pathways relevant to the PCOS–periodontitis linkage, thus highlighting key molecular mechanisms that may be associated with the development of the two diseases. Genes such as ACSL5, NLRP12, CCRL2, and CEACAM3 were identified as the molecular link between PCOS and periodontitis. These findings provide a molecular basis for future experimental studies on the association between PCOS and periodontitis.

### Supplementary Information

**Additional file 1: Figure 1.** Removal of batch effect in the PCOS and periodontitis training datasets. **1A.** PCA of PCOS before batch effect correction; **1B.** PCA of PCOS after batch effect correction with ComBat;**1C.** PCA of periodontitis before batch effect correction with ComBat; **1D.** PCA of periodontitis after batch effect correction with ComBat. Blue triangles in 1A and 1B denote PCOS. Blue triangles in 1C and 1D denote periodontitis. Red dots in 1A, 1B, 1C, and 1D denote normal samples.

**Additional file 2: Figure 2.** Functional analysis of intersected genes involved in PCOS and periodontitis. **2A.** PPI network of 40 interacted genes, the colors of circles represent different Log (fold change) values;**2B.** Go enrichment analysis of intersected genes, different colors in the figure represent different P values,according to the significance threshold P.value < 0.05; **2C.** KEGG pathway enrichment analysis of intersected genes, different colors in the figure represent different P values, according to the significance threshold P.value < 0.05; **2D.** The gene regulation network, hexagons represent common genes, circles represent common pathways. Different colors represent different Log (fold change) values.

**Additional file 3: Figure 3.** ROC curves of hub genes expression in PCOS-training dataset. The area under the ROC curve represents the AUC value.

**Additional file 4: Figure 4.** ROC curves of hub genes expression in periodontitis-training dataset. The area under the ROC curve represents the AUC value.

**Additional file 5: Figure 5.** TF-ceRNA network of diagnostic genes. **5A**. TF-diagnostic gene network;**5B**. ceRNA network of diagnostic genes; **5C**. TF-ceRNA network of diagnostic genes.

**Additional file 6: Table S1.** Primer sequences for PCR.

**Additional file 7: Table S2.** Trends in intersected genes expression.

**Additional file 8: Table S3.** Topology structure of the PPI network.

**Additional file 9: Table S4.** Results of GO enrichment.

**Additional file 10: Table S4.** Results of GO enrichment.

**Additional file 11: Table S6.** CeRNA network of diagnostic genes.

## Data Availability

The datasets generated and/or analyzed during the current study are available in the GEO repository (https://www.ncbi.nlm.nih.gov/geo/).
