# Supplementary figures and images for: Transcriptomic analysis identifies diagnostic genes in polycystic ovary syndrome and periodontitis
Source: Eur J Med Res. 2024 Jan 2;29:3. doi: 10.1186/s40001-023-01499-4 (PMC10762819; doi:10.1186/s40001-023-01499-4)

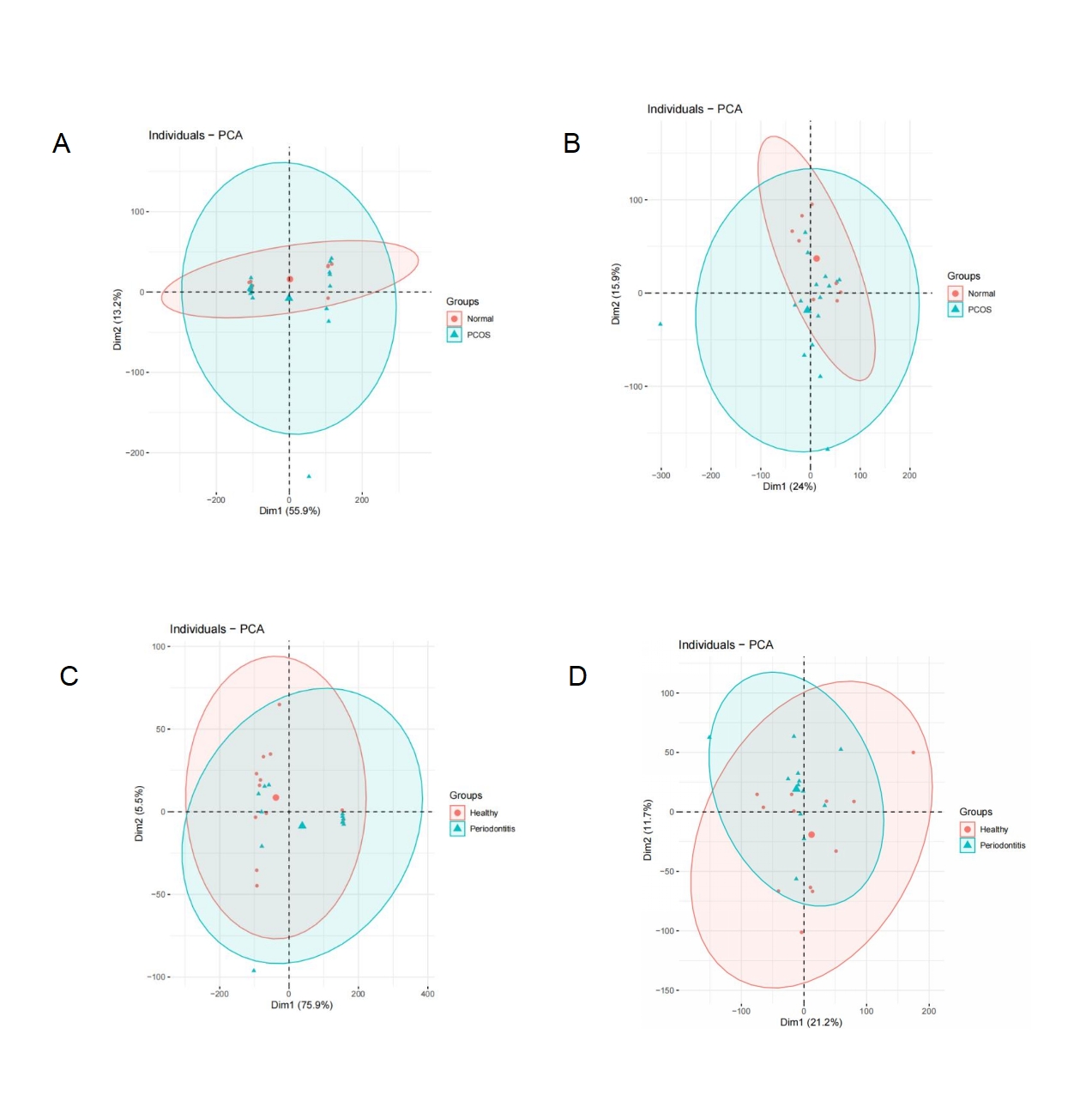

Supplement: Supplementary file 1 — Additional file 1: Figure 1. Removal of batch effect in the PCOS and periodontitis training datasets. 1A. PCA of PCOS before batch effect correction; 1B. PCA of PCOS after batch effect correction with ComBat;1C. PCA of periodontitis before batch effect correction with ComBat; 1D. PCA of periodontitis after batch effect correction with ComBat. Blue triangles in 1A and 1B denote PCOS. Blue triangles in 1C and 1D denote periodontitis. Red dots in 1A, 1B, 1C, and 1D denote normal samples. [file 40001_2023_1499_MOESM1_ESM.jpg]

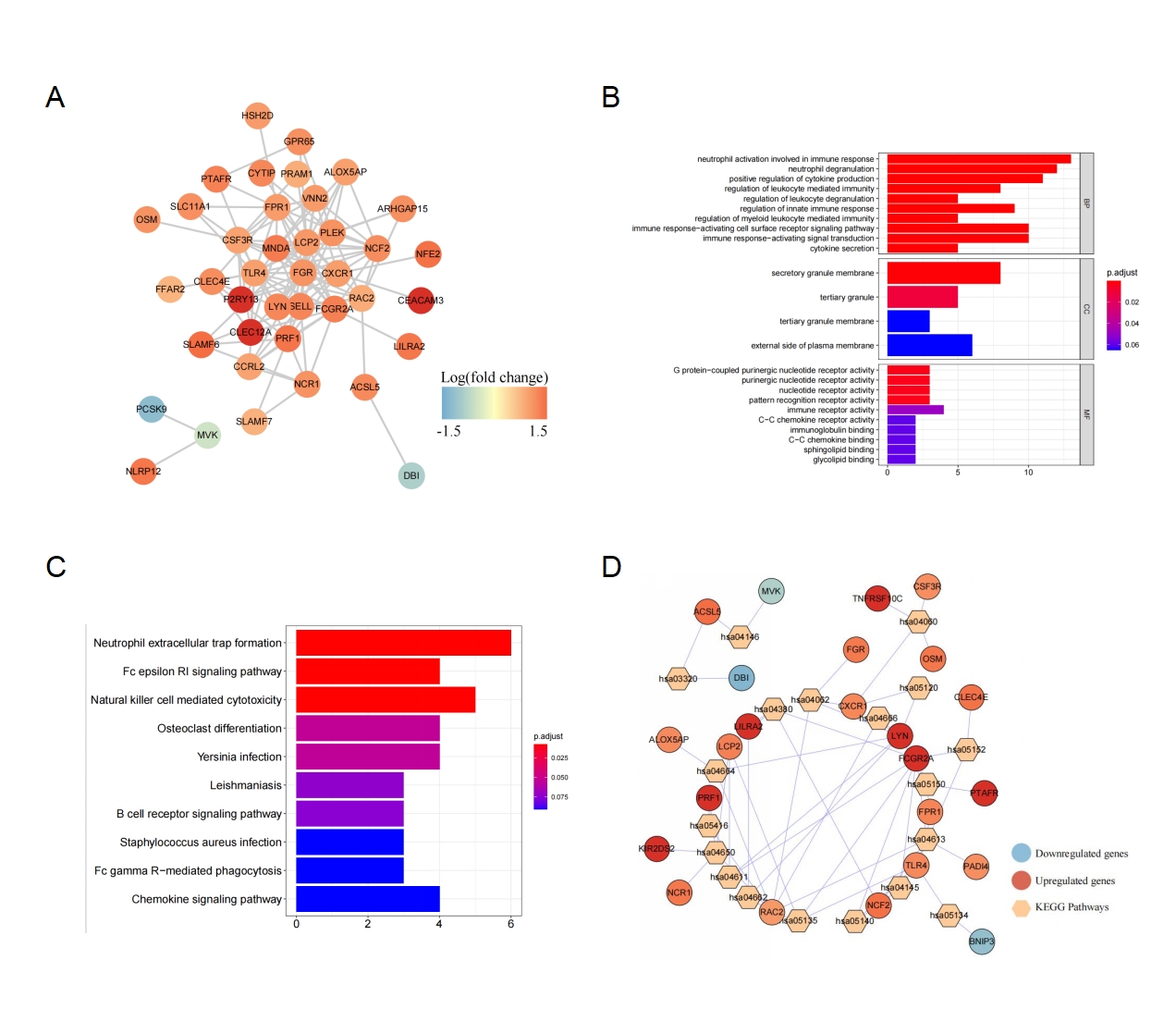

Supplement: Supplementary file 2 — Additional file 2: Figure 2. Functional analysis of intersected genes involved in PCOS and periodontitis. 2A. PPI network of 40 interacted genes, the colors of circles represent different Log (fold change) values;2B. Go enrichment analysis of intersected genes, different colors in the figure represent different P values,according to the significance threshold P.value < 0.05; 2C. KEGG pathway enrichment analysis of intersected genes, different colors in the figure represent different P values, according to the significance threshold P.value < 0.05; 2D. The gene regulation network, hexagons represent common genes, circles represent common pathways. Different colors represent different Log (fold change) values. [file 40001_2023_1499_MOESM2_ESM.jpg]

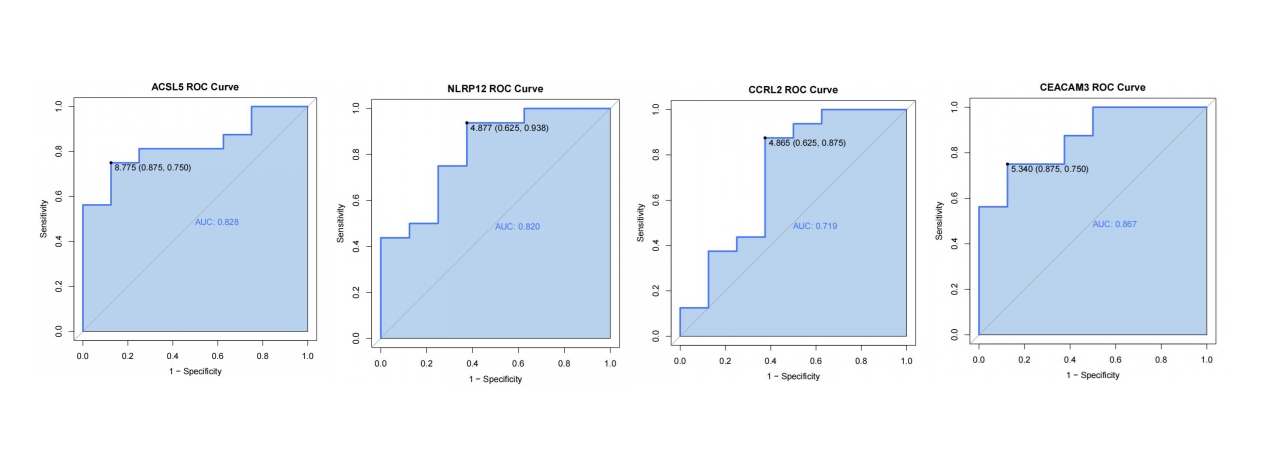

Supplement: Supplementary file 3 — Additional file 3: Figure 3. ROC curves of hub genes expression in PCOS-training dataset. The area under the ROC curve represents the AUC value. [file 40001_2023_1499_MOESM3_ESM.jpg]

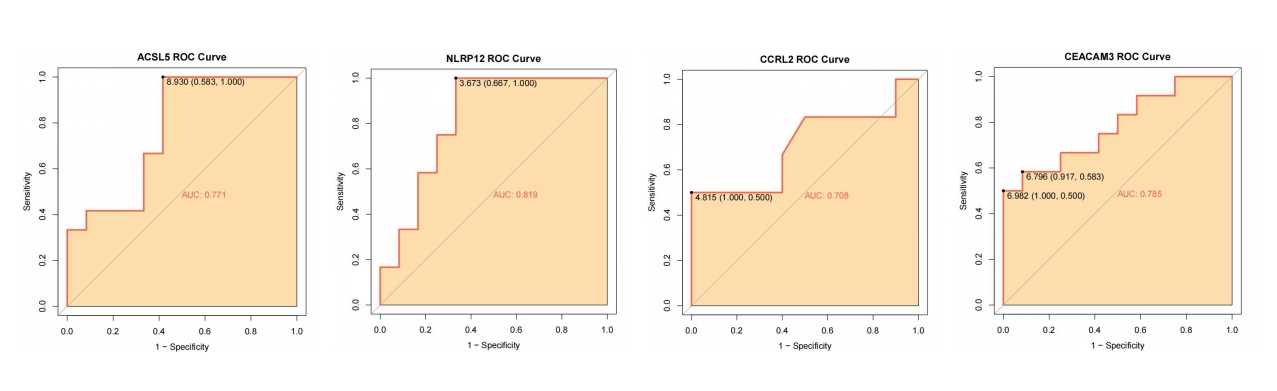

Supplement: Supplementary file 4 — Additional file 4: Figure 4. ROC curves of hub genes expression in periodontitis-training dataset. The area under the ROC curve represents the AUC value. [file 40001_2023_1499_MOESM4_ESM.jpg]

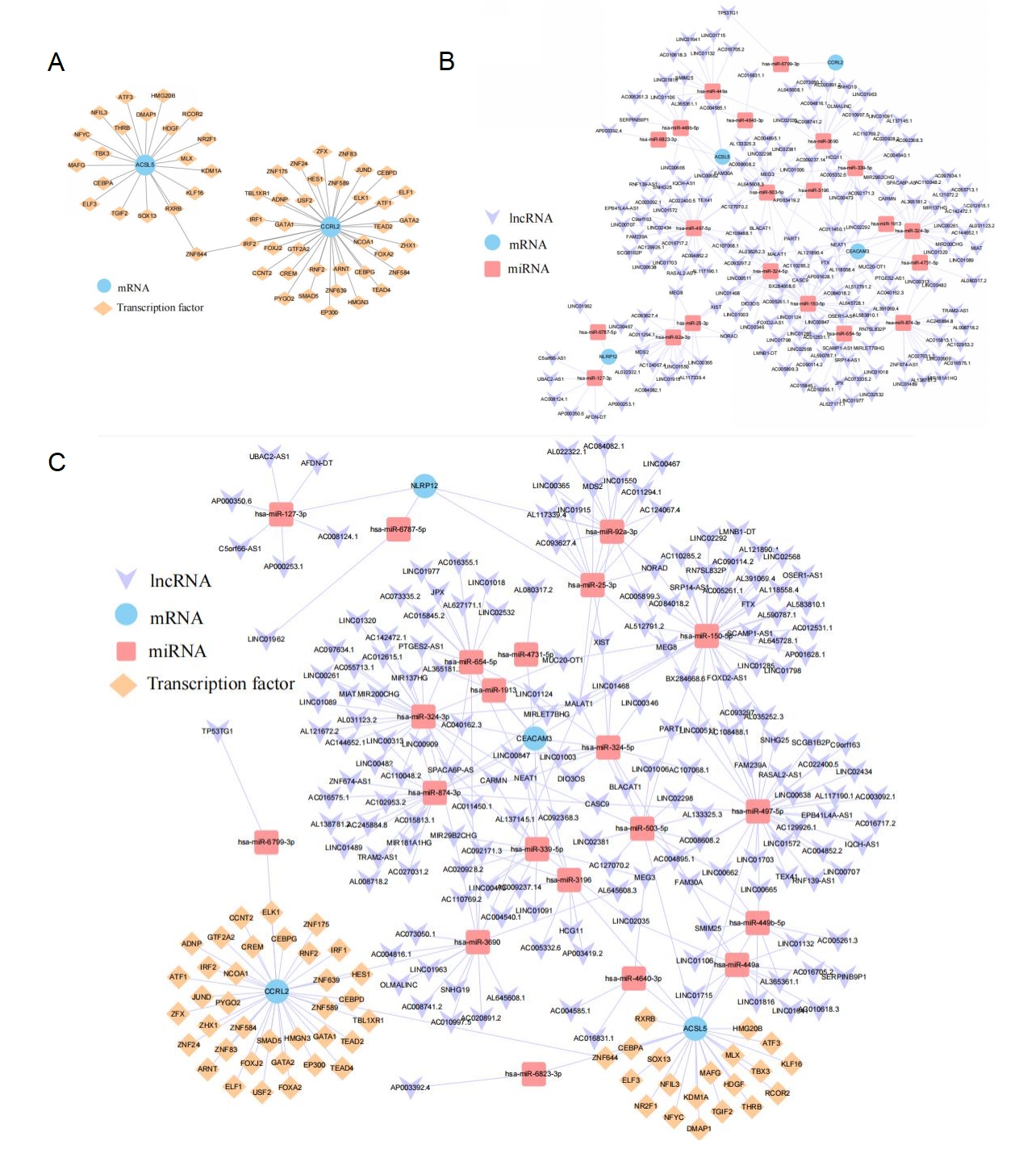

Supplement: Supplementary file 5 — Additional file 5: Figure 5. TF-ceRNA network of diagnostic genes. 5A. TF-diagnostic gene network;5B. ceRNA network of diagnostic genes; 5C. TF-ceRNA network of diagnostic genes. [file 40001_2023_1499_MOESM5_ESM.jpg]
